# Deleted in liver cancer 1 expression and localization in hepatocellular carcinoma tissue sections

**DOI:** 10.3892/ol.2014.2216

**Published:** 2014-06-03

**Authors:** DOMINIKA WOLOSZ, AGNIESZKA WALCZAK, GRZEGORZ M. WILCZYNSKI, GRZEGORZ SZPARECKI, EWA WILCZEK, BARBARA GORNICKA

**Affiliations:** 1 Department of Pathomorphology, Medical University of Warsaw, Warsaw, Mazovia 02-091, Poland; 2 Laboratory of Molecular and Systemic Neuromorphology, Nencki Institute of Experimental Biology, Polish Academy of Sciences, Warsaw, Mazovia 02-093, Poland

**Keywords:** deleted in liver cancer 1, hepatocellular carcinoma, fibrolamellar carcinoma

## Abstract

The deleted in liver cancer (DLC) protein family is composed of proteins that are hypothesized to function predominantly by regulating the activity of the small GTPases. The aim of the present study was to determine the expression and exact localization of DLC1 in hepatocellular carcinoma (HCC) tissue sections. In two types of HCC tissues, typical and fibrolamellar, immunohistochemical and immunofluorescent analysis were performed to assess DLC1 immunoreactivity. Additionally, the *DLC1* gene status was determined by the fluorescence *in situ* hybridization. According to the observations, DLC1 is often lost in cancer cells; however, it can remain within the stromal component of tumor sections. The DLC1 immunoreactivity was particularly noticeable within the capsules surrounding the tumor masses. It was found that the *DLC1* gene was deleted in 52% of HCC cases. In addition, the hemizygous deletion was observed to be independent of the HCC subtype. The results indicate that although the loss of DLC1 is a common step during hepatocarcinogenesis, this protein may be present in the tumor microenvironment.

## Introduction

The deleted in liver cancer 1 *(DLC1)* gene was initially identified to be deleted in primary hepatocellular carcinoma (HCC) in 1998 (1). Subsequent studies have identified its frequent loss of function as a consequence of gene deletion or epigenetic silencing in numerous malignancies (2–4). *DLC1* is located on chromosome 8p21-22 and encodes a Rho GTPase-activating protein (GAP), a negative regulator of the Rho family of small GTPases. The typical structure of a DLC1 protein is composed of three functional domains: Sterile-α-motif (SAM), RhoGAP and steroidogenic acute regulatory-related lipid transfer. Since RhoGAPs are significant regulators of Rho proteins, including RhoA or CDC42, the function of DLC1 is primarily linked to the regulation of the actin cytoskeleton organization, formation of actin stress fibers, and focal adhesions and cell cycle progression. The tumor suppressor function is predominantly associated with its RhoGAP activity, although other aspects of the protein, such as the SAM domain, may also regulate this activity (5,6).

HCC is the most common primary liver cancer and the fifth most common cancer in the world (7). The treatment methods remain insufficient and the overall prognosis is poor due to the high tumor recurrence, resistance to anticancer agents and ineffective chemotherapy in patients with underlying cirrhosis and impaired liver function. Thus far, surgical intervention remains the most effective treatment, however, 70% of HCC patients are unable to receive this type of therapy due to their tumors exhibiting an advanced stage when subjected to treatment (8). Currently there are few biomarkers that may be potential therapeutic molecules. A multikinase inhibitor, sorafenib, was the first promising therapeutic agent designed for HCC treatment, however, its efficacy requires improvement (9).

The fibrolamellar (FL) variant of HCC represents a rare form of this primary liver cancer and is typical in young individuals. It usually develops in the liver without underlying cirrhosis or other concurrent liver disease (10). The molecular changes associated with the two variants of HCC are currently poorly understood.

The aim of the present study was to determine the presence and tissue/cellular localization of the DLC1 protein in HCC tumor samples. Additionally, the genetic probe for *DLC1* was designed (using an *in situ* hybridization technique) to identify whether the loss of this gene is a common change during the development of typical and the FL variant of HCC.

## Materials and methods

### Tissue specimens

The tissue samples were obtained from 81 patients diagnosed with HCC and a total of nine exhibited FL tumors. The control group comprised of 25 specimens of normal liver tissue.

### Ethics statement

The present study was approved by the Medical University of Warsaw (Warsaw, Poland) Ethics Committee (KBO/42/11).

### Immunohistochemistry and immunofluorescence

Formalin-fixed, paraffin-embedded sections were deparaffinized, blocked with 3% hydrogen peroxide and 5% normal donkey serum (Jackson Immunoresearch Labs, Inc., West Grove, PA, USA). The sections were pretreated with a pepsin digestion. The DLC1 immunoreactivity was determined using the goat polyclonal anti-DLC1 primary antibody (Abcam, Cambridge, United Kingdom). The detection was performed with a donkey anti-goat biotin-conjugated antibody (Jackson Immunoresearch Labs, Inc.) followed by an ABC Vectastain kit (Vector Laboratories, Burlingham, CA, USA) and 3,3′-diaminobenzidine (DakoCytomation, Glostrup, Denmark) as a chromogen. For histochemical staining of the reticulin fibers, routine procedures were conducted (11).

For immunofluorescence studies, the primary antibodies were detected by secondary donkey anti-goat and donkey anti-mouse polyclonal antibodies with Alexa Fluor^®^ 555 and −488 dyes (Jackson Immunoresearch Labs, Inc.). For colocalization analysis the monoclonal mouse anti-cluster of differentiation (CD)34 (DakoCytomation), polyclonal rabbit anti-hepatocytes (Biogenex, Fremont, CA USA) and monoclonal mouse anti-collagen IV (Leica Microsystems, Mannheim, Germany) antibodies were applied. The sections were mounted using Vectashield with 4′,6-diamidino-2-phenylindole (Vector Laboratories). Immunofluorescence was analyzed under a Leica TCSSP5 confocal microscope (Leica Microsystems).

### Fluorescence in situ hybridization probe preparation

The *DLC1* sequence was obtained from the bacterial artificial chromosome DNA library (Children’s Hospital Oakland Research Institute, Oakland, CA, USA) as a bacterial lysogeny broth (LB) agar stab culture. *Esherichia coli* were cultured in LB agar with chloramphenicol, passaged as a single isolated colony and subjected to a rapid alkaline-DNA isolation by the EndoFree Plasmid Maxi kit (Qiagen, Hilden, Germany). The extracted DNA was amplified using a GenomiPhi DNA Amplification kit (GE Healthcare, Pittsburgh, PA, USA). Subsequently, deoxyuridine triphosphate was labeled with digoxygenin or the biotin Translation mix (Roche Applied Science, Indianapolis, IN, USA) and the probe was labeled by the nick-translation method according to Cremer *et al* (12).

### Hybridization procedure using a modified Cremer et al (12) method

For the hybridization procedure, sections were deparaffinized and hydrated in a series of alcohol. Subsequently, digestion with pepsin and tissue permeabilization with sodium thiocyanate at 80°C was performed. The sections were equilibrated in 50% formamide/2X saline-sodium citrate (SSC), pre-hybridized in 45°C, denatured in 80°C and hybridized for 40 h. In brief, post-hybridization procedures were performed by incubation of the sections in 2X SSC and 0.1X SSC in 60°C for 2×10 min. Detection of the biotin-conjugated probes was performed by incubation with avidin-Alexa Fluor^®^ 488 (Molecular Probes, Life Technologies, Inc., Eugene, OR, USA) and anti-avidin-fluorescein-isothiocyanate (Sigma Aldrich, St. Louis, USA) antibodies. For visualization of the nuclei, the sections were stained with Hoechst (Molecular Probes, Life Technologies, Inc.). The specimens were examined under a Nikon Eclipse 80i fluorescence microscope (Nikon, Tokyo, Japan). To assess the gene status in each section, 200 cells were counted (magnification, ×600).

## Results

### DLC1 immunoreactivity predominantly localizes in the stromal component of HCC tissue

**Figure 1:**
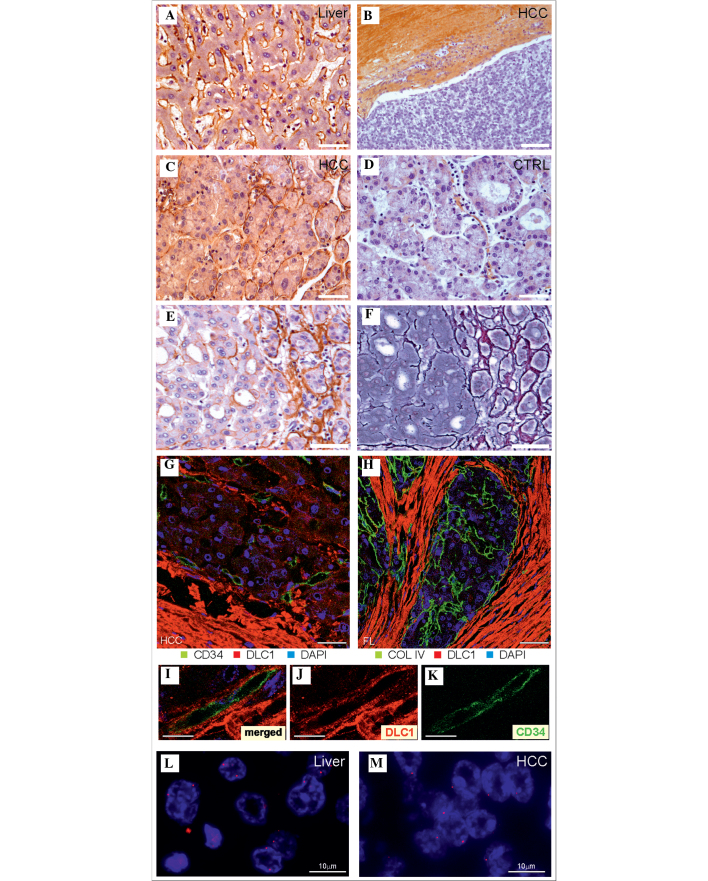
DLC1 immunoreactivity in normal liver and in HCC tissue sections. (A) Staining in the normal liver; (B) the cumulated immunoreactivity at the periphery of the HCC tissue and the capsule of the tumor; (C) HCC representing moderate staining; (D) control immunoreaction; (E and F) the comparison between (E) DLC1 immunoreactivity and (F) the reticulin fiber staining on serial sections of the HCC tumor sample; (G, I-K) combined immunofluorescence of DLC1 (red) and CD34 (green); note the DLC1 immunoreactivity localized adjacent to the endothelial cell; (H) immunofluorescent staining with the anti-DLC1 antibody (red) and anti-type IV collagen (green); note completely separate localization; (L and M) representative results of the fluorescence *in situ* hybridization with the DLC1 probe (L) in the normal liver and (M) in HCC tissue. Scale bars: A, C and D, 50 μm; B, 100 μm; E and F, 30 μm; G and H, 20 μm; I-M, 10 μm. DLC1, deleted in liver cancer 1; HCC, hepatocellular carcinoma; CTRL, control; CD34, cluster of differentiation 34; DAPI, 4′,6-diamidino-2-phenylindole; COL IV, collagen IV.

Figure 1A–E demonstrates DLC1 immunoreactivity in normal liver and HCC samples. In the normal liver, DLC1 immunoreactivity was found to primarily localize in the periportal zone of hepatic lobules and in sinusoids (Fig. 1A). In hepatocytes, weak cytoplasmic immunoreactivity was detected.

In 34 out of 81 HCC tissues, no DLC1 immunoreactivity was observed in the cytoplasm of the cancer cells (Fig. 1C). In the remaining tumor samples, DLC1 was observed as a weak or moderate staining of the cytoplasm of the cancer cells. By contrast, in stromal components, and particularly in the tumor capsules surrounding the tumor masses, a strong DLC1 immunoreactivity was observed in virtually all of the examined samples (Fig. 1B, G and H). To identify whether DLC1 is associated with endothelial cells or basal lamina, double immunofluorescence studies were performed with anti-DLC1, and anti-CD34 and anti-type IV collagen antibodies, respectively. Although, DLC1 did not appear to colocalize exactly with either structure (Fig. 1G and H), its immunoreactivity was concentrated within a close proximity to the endothelial cells (Fig. 1I–K). The FL variant of HCC exhibited a strong DLC1 immunoreactivity that was localized in the bundles of the connective tissue surrounding the tumor cells (Fig. 1H). The negative control did not exhibit immunoreactivity (Fig. 1D).

The comparison between the immunohistochemical DLC1 staining and histochemical reticulin fiber staining on serial sections revealed comparable patterns of localization (Fig. 1E vs. F). A lack of DLC1 immunoreactivity was observed to localize primarily within the sites of tumor samples that demonstrated diminished reticulin fiber staining.

### A single allele of the DLC1 gene is frequently lost in HCC

The fluorescent *in situ* studies with probes designed for the *DLC1* gene showed a frequent detection of one copy-number of the *DLC1* gene in HCC (Fig. 1L and M). A deletion was detected in 13 of the 25 (52%) tumors that were studied. This genetic change generally coincided with the loss of protein expression, although in a population of tumors, despite the presence of the *DLC1* alleles, the immunoreactivity was completely diminished. The gene deletion was found to be independent of the HCC subtype.

## Discussion

The presence and localization of the DLC1 protein in HCC is demonstrated in the present study. The results identified that the *DLC1* gene was deleted in a high percentage of the examined sections. It has been demonstrated for the first time, to the best of our knowledge, that the DLC1 protein, although lost in cancer cells, can be present in the tumor microenvironment, which is particularly visible in the FL variant of HCC.

The first study concerning *DLC1* function was performed in animal models and showed that deficiency of this gene leads to embryonic lethality (13). A subsequent study based on the comparative hybridization technique demonstrated that the chromosomal region spanning *DLC1* (8p21) shows frequent deletions in various types of cancer (3). Thus far, its role as a protein that affects tumorigenesis has been documented in neoplasms, such as lung and breast cancer, Burkitt’s lymphoma, clear cell renal carcinoma and meningioma (14–18). As shown in the study by Wong *et al* (19), gene copy loss or epigenetic gene silencing are responsible for the underexpression of DLC1 in HCC.

The results of the present study are generally consistent with the aforementioned studies, as the loss of the DLC1 immunoreactivity was observed within cancer cells. Notably, it was found in the examined tissue samples that the DLC1 immunoreactivity was present in the other tissue components of the normal liver and HCC sections. The issue of the role of the stromal component in liver cancer development has been raised in a recent study (20). Numerous cell types, including carcinoma-associated fibroblasts, activated hepatic stellate cells, tumor-associated macrophages and endothelial cells, or dendritic cells were found to affect cancer cell growth, invasion, vasculature formation or metastatic capabilities. The effect of the stromal cells on HCC development may be executed in various ways. Tumor-associated macrophages were shown to promote metastasis and angiogenesis through the release of various growth factors (21). A study by Amann *et al* (22) showed that activation of the hepatic stellate cells may promote tumorigenesis through the activation of nuclear factor-κB and extracellular-regulated kinase in HCC cells. Additionally, the stellate cells and macrophages, when activated, are a potent source of osteopontin (23). The role of this protein in the remodeling of the tumor microenvironment and metastatic process was well documented in numerous types of cancer (24). In HCC the increased level of this protein correlates with a higher potential of invasiveness (25). As was shown in the study by Zhou *et al* (26), the restoration of the DLC1 protein in the cancer cell lines, which were previously devoid of DLC1, reduces the expression of mRNA for osteopontin, which indicates that DLC1 possesses the ability to negatively regulate osteopontin. Furthermore, analogous regulation was observed regarding the mRNA for matrix metalloproteinase 9, an enzyme with a well-known function in tumor progression. Thus, localization of DLC1 in the tumor stroma or tumor capsule functions as an inhibitor of the extracellular matrix degradation, which prevents the spreading of cancer cells. In conclusion, in the present study, DLC1 was found to be abundantly present in the environment of the FL variant of HCC, which is rich in fibrous components; furthermore, the FL variant is considered to be a slowly proliferating tumor. Therefore, the low proliferation rate may be associated with the abundant presence of the tumor suppressor, DLC1 within the tumor microenvironment.
